# Use of pharmacy dispensing data to measure adherence and identify nonadherence with oral hypoglycemic agents

**DOI:** 10.1007/s00228-016-2149-3

**Published:** 2016-10-28

**Authors:** Fong Sodihardjo-Yuen, Liset van Dijk, Michel Wensing, Peter A. G. M. De Smet, Martina Teichert

**Affiliations:** 1 Royal Dutch Pharmacists Association, 2514 JL The Hague, the Netherlands; 2 Netherlands Institute for Health Services Research, Utrecht, the Netherlands; 3 Radboud Institute for Health Sciences, Department of IQ Healthcare, Radboud University Medical Centre, Nijmegen, the Netherlands; 4 Radboud Institute for Health Sciences, Department of Clinical Pharmacy, Radboud University Medical Centre, Nijmegen, the Netherlands; 5 Department of Clinical Pharmacy & Toxicology, Leiden University Medical Center, Leiden, the Netherlands

**Keywords:** Adherence calculation, Nonadherence, Pharmacy dispensing data, Oral hypoglycemic agents

## Abstract

**Purpose:**

A framework for calculation of adherence for oral hypoglycemic agents (OHAs) based on data from health-insurance claims is available. Pharmacy dispensing data aid identification of nonadherent patients in pharmacy practices. However, use of these data for calculation of OHA adherence requires additional methodological categories. We examined the impact of different methodological choices on estimation of OHA adherence using pharmacy dispensing data.

**Methods:**

Four methodological categories were added to the framework available to be used for adherence calculation with pharmacy dispensing data. Three adherence measures were defined to supply pharmacists with significant information on OHA use of their patients: (i) percentage of days covered by use periods of dispensed medication (PDC), (ii) mean rate of adherent patients with a PDC ≥80 % (MRAP80), and (iii) mean number of nonadherent patients (MNNP80) per pharmacy with a PDC <80 %. A basic scenario was developed from 16 methodological categories. Consequences of choices for different parameters within these categories on the scores of the three adherence measures were calculated from dispensing data between July 2013 and July 2014.

**Results:**

Data were available for 604,500 OHA users in 1737 community pharmacies in the Netherlands. For the basic scenario, mean PDC for OHA was 88.3 %. MRAP80 was 80.3 %, which corresponded to an average of 69 nonadherent patients per pharmacy. Different choices for parameter values resulted in score variations for PDC of 85.0–91.8 %, for MRAP80 of 75.3–86.1 %, and between 49 and 92 MNNP80 per pharmacy.

**Conclusion:**

Sixteen methodological categories specified calculation of OHA adherence based on pharmacy dispensing data. Adherence scores expressed as percentages were relatively robust to variation in parameter values, but differed substantially for the absolute numbers of nonadherent patients per pharmacy.

**Electronic supplementary material:**

The online version of this article (doi:10.1007/s00228-016-2149-3) contains supplementary material, which is available to authorized users.

## Introduction

Nonadherence to prescribed medication has been identified to be a major source of suboptimal management of several chronic diseases [1–5]. A recent Cochrane review reported that prescription-refill records collected routinely are a more objective method to measure adherence than self-reporting by patients, and less expensive than Medication Event Monitoring Systems [6]. Based on prescription-refill records, adherence can be measured as average medication availability by the percentage of days covered by drug use periods of dispensed medication (PDC) or the mean rate of adherent patients for a certain threshold, chosen mostly at PDC ≥ 80 % (MRAP80). For calculation of these measures, several methodological choices must be made. Dependent on these choices, a large variation in corresponding adherence scores has been reported [7–13].

Transparency in calculation of adherence scores is needed to assess the validity of results and to enable comparisons between different studies [14–18]. Wilke et al. defined 12 methodological categories and analyzed the influence of variation in parameter values within these categories on the prevalence of nonadherence for oral hypoglycemic agents (OHAs) with claims data from a health-insurance fund in Germany [7]. These categories addressed choices in patient inclusion (e.g., based on the diagnosis, a certain number of prescriptions, and new or prevalent users), duration of the observation period, and period of drug use. They dealt with periods of medication cessation and switching within drug classes, stockpiling, overuse and the threshold of minimum PDC to define nonadherence [7]. Wilke et al. showed that some parameters had a stronger effect on adherence scores than others. In particular, the availability of information on the prescribed daily doses (PDDs) used to calculate periods of drug use changed PDC by 25 % [7]. Information on the PDD and the number of tablets dispensed can be used to calculate periods of drug use more accurately than counting the number of dispensations. Usually, information on PDDs is available from clinical prescribing or dispensation records, but is not always present in databases for health-insurance claims. Another advantage of pharmacy dispensing data is that they enable pharmacists to identify patients for additional pharmaceutical care early as those who do not refill medication for their chronic disease in time. Community pharmacists have been shown to improve nonadherence to therapies for chronic disease using their dispensing data [19–23]. However, pharmacy dispensing data have a limitation in that adherence scores for patients are usually calculated from dispensations within individual pharmacies only and do not allow following up of patients for dispensations across different pharmacies (which is possible from health-insurance claims). Consequently, additional methodological categories are needed to identify patients with dispensations in several pharmacies (“drop-in” patients) and those who cease to visit the pharmacy because of a change of address, hospital admission, or death (“without any dispensations in a certain time period”). For OHA adherence, an additional methodological category for dealing with insulin besides OHA use is also helpful. According to guidelines, initiation of insulin usually involves cessation of all but one OHA used previously. Thus, a reduction in OHA use in combination with insulin initiation could be assessed incorrectly as “patient nonadherence.” For implementation of additional pharmaceutical care to nonadherent patients in pharmacies, information on the absolute number of patients identified as being nonadherent per pharmacy is also important.

These theoretical considerations gave rise to develop additional methodological categories to calculate adherence scores for OHAs using pharmacy dispensing data.

The main objective of the present study was to calculate OHA adherence based on a methodological framework for three measures: (i) PDC, (ii) mean percentage of patients with a PDC ≥80 (MPAP80), and (iii) mean number of nonadherent patients at a PDC <80 % (MNNP80) per pharmacy. In addition, the consequences of alternative methodological choices on the scores of these adherence measures were calculated.

## Methods

### Ethical approval of the study protocol

Data from pharmacists and patients were coded and anonymized before analyses. Use of observational data in descriptive retrospective studies in the Netherlands is not considered as an interventional trial according to Directive 2001/20/EC and Dutch legislation [24]. Therefore, the study protocol did not need to be submitted to a medical ethic committee for approval.

### Data sources

In the Netherlands, OHAs are available by prescription only. All dispensations are registered in the computer system of the pharmacy per patient for health-insurance claims as well as for automated surveillance of medication use. Dispensing data from more than 90 % of the 1981 community pharmacies in the Netherlands are delivered every month to the Foundation of Pharmaceutical Statistics (SFK) on a routine basis. These data provide detailed information on the drugs dispensed, including the codes from the Anatomic Therapeutic Chemical (ATC) system of the World Health Organization [25], the prescribed daily dosage (PDD), and the amount dispensed. From this information, periods of drug use can be calculated by dividing the amount dispensed by the PDD. Data on patient information in the SFK consist of an anonymous number from the computer system of the local pharmacy and information on the patient’s sex and year of birth.

### Data collection

Data were collected from the SFK for community pharmacies that had provided complete dispensing data from 1 July 2012 to 1 July 2014. This period covered the study period from 1 July 2013 to 1 July 2014. The data history for the year before the study period was needed to state first OHA dispensings as those dispensings within the study period without a dispensing in the year before. From the community pharmacies included, patients who used at least one OHA (ATC-code A10B) within the study period were eligible to be included in adherence calculations. Medication could be tracked for a specific patient over time by a unique anonymous patient code within an individual pharmacy. Pharmacies with shared populations and using the same anonymous codes for their patients could label themselves as “pharmacy clusters” at SFK. In these specific cases, dispensations for these patients could be followed up across pharmacies within a cluster.

### Outcome measures

Two adherence measures were calculated. The first was the PDC and was the average number of days covered by drug use periods of dispensed medication in relation to all days during the study period (calculated from the first day of drug use until the end of this period as a medication possession rate) [7, 26]. The second measure was MRAP80 as the rate of OHA user with a PDC ≥80 % within all OHA users in the study period. An additional outcome measure for the potential effort for the pharmacists to provide additional care for their nonadherent patients was defined. This is stated as the mean absolute number of nonadherent patients at a PDC <80 % (MNNP80) per pharmacy within the pharmacies included.

### Methodological categories to measure adherence using pharmacy dispensing data

Four methodological categories were added to the methodological framework with 12 categories for data on health-insurance claims as defined by Wilke et al. [7] as categories 13–16 (Table 1). Three categories were added with regard to the use of dispensing data per pharmacy for: drop-in patients; “actual patients” (patients with recent dispensations to ensure that patients continue to visit a particular pharmacy and did not, for example, move away or die); the possibility to follow-up patients for their dispensations from several community pharmacies within pharmacy clusters. A fourth category was added whether to include OHA users who also used insulin.

**Table 1:** Methodological categories to calculate adherence and identify nonadherence from pharmacy dispensing data

Category^a^	Basic case	Related parameters	Considerations for pharmacy dispensing data
1. Drug prescribing, dispensing or diagnosis as inclusion criterion	Patient selection based on at least one dispensing of ATC^b^ drug class A10B within the study period	*Number, source of diagnosis* ^c^	As pharmacy dispensing data lack information on diagnoses, diseases have to be estimated by dispensings. As oral hypoglycemic agents (OHA) are specifically used for type 2 diabetes mellitus, dispensing data can be used to select diabetes patients. If information on diagnoses is available, the source can be used for more valid patient selection, e.g., specialist diagnosis
2. Patient type as inclusion criterion	All patients using at least one dispensing of ATC class A10B within the study period, based on a dispensing in the study period or the 3 months prior	a) Only prevalent users are included in adherence calculation, new users are excluded b) Users that stopped with use of their medication are excluded c) *Only newly treated patients are included*	a) New users are defined as subjects with an OHA dispensing during the study period and no dispensing of any OHA drug during the prior 12 months b) Stoppers are defined as subjects without a refill within the last 4 months of the study period. This excluded moved or ceased patients. The period of 4 months was chosen prescriptions generally are dispensed for 3 months c) *To choose when specific attention is paid to starting drug use*
3. Minimum number of prescriptions as inclusion criterion	At least one dispensing during the study period	At least two or more dispensings in the study period	A higher number of dispensings could indicate that the therapy is chronic or that the patient is a permanent client of a community pharmacy. However, in nonadherence calculation, criteria for a higher number of dispensings induce bias by excluding patients who are not adherent after a small number of dispensings. To define permanent (no “drop-in”) and actual pharmacy clients, other parameters can be used (see category 14 and 15)
4. Observation period	Annual analysis: July 2013–July 2014	*Analysis for several months or years*	In the literature, observational periods of several months up to 10 years are used
5. Period of drug use	Based on drug supply and prescribed daily doses	*Based on a minimum number of dispensings*	Pharmacy dispensing data offer information on the prescribed daily dose (PDD). Thus periods of drug use can be calculated for distinct pharmaceutical formulations such as oral drugs by dividing the total number of dispensed drug units by the PDD. Another, more basic way, for calculating nonadherence is to consider a patient as adherent when a specified number of prescriptions is refilled during the study period. Alternative methods of defining drug use periods, like what Wilke et al. applied, are based on the defined daily dose (DDD) or guideline recommendations. These approaches are however less accurate and useless when the PDD is available
6. Time interval under observation	Interval-based from the first dispensing until the end of the study period	*Dispensing-based (denominator: first until last dispensing, numerator: dispensed doses at the first until the one but least dispensing)*	With an interval-based nonadherence measure, the end of the interval is taken as a fixed date. This implicates that periods of drug discontinuation are included in the adherence measures. In dispensing-based calculation, discontinuation of therapy is not measured. To monitor treatment adherence of their patients, pharmacists also have to elucidate the reasons for treatment cessation, e.g., intentional, in agreement with the prescriber. Thus, a dispensing-based approach is not sufficient for pharmacists interventions
7. Adherence measure: assumption of concomitant use or switching between drugs/drug classes	Concomitant use considered for different drug classes: estimation of the percentage of days covered with medication (PDC) separately calculated for the different OHA drug classes in use by one patient as arithmetic mean of the ATC group-specific PDCs	Switching considered between drug classes: estimation of the PDC as percentage of days covered by any OHA drug class	When assessing the PDC, it has to be decided on which ATC class level coverage of drug use is considered. This decision implies whether a patient is still adherent when switching to another drug, e.g., from metformin from one brand to another or also when switching from one OHA drug class to another (e.g., from metformin to glibenclamide). As in T2DM therapy, several OHA classes are used concomitantly, and thus metformin may rather have to be used concomitantly with glibenclamide
8. Stockpiling	Stockpiling considered	No stockpiling considered	When accounting for stockpiling, periods of drug use are adjusted for early refills
9. Dealing with adherence >100 % on patient level	Truncation to 100 %	*No truncation to 100 %*	With truncation, only nonadherence periods of underuse are considered with no periods of overuse. For OHA, overuse does not seem an issue
10. Number of analyzed medication classes	Adherence measures for all medication classes for patients with mono- and multimedication, within OHA	*Inclusion of only patients with monomedication or only analysis of one drug*	If adherence has to be calculated for a specific drug (class), adherence measures could be calculated for instance for metformin only within patients using metformin only or also additional OHA drugs. For pharmacists’ interventions, the whole diabetes medication has to be taken into account. Thus, focus on one drug is not useful
11. Absence periods (e.g., hospital stay, holiday)	Exclusion of patients with absence periods	Inclusion of patients with absence periods	When measuring adherence from the dispensings from one pharmacy only, patients might be falsely classified as nonadherent because of dispensings from other pharmacies, at hospital or during vacation. Periods of drug supplies from elsewhere are clearly visible in dispensing maps for patients using several chronic medications as simultaneous gaps in drug use. Patients with such gaps in their medication profiles can be excluded from adherence calculations to warrant the measures based on valid information
12. Threshold to calculate the mean rate of adherence patients	Threshold for nonadherence at an PDC <80 %	Threshold for nonadherence at PDC <60 or <90 %	Some drugs are more and others are less “forgiving” for doses missed. According to drug class characteristics, the nonadherence threshold should be accustomed. A threshold of 80 % is most common
13. “Drop-in” patients as exclusion criterion	Exclusion of “drop-in” patients	Inclusion of “drop-in” patients	“Drop-in” patients are defined as those with only one or two dispensings within four consecutive days of any ATC drug class within 2 years. Although the fidelity of Dutch patients to one community pharmacy is high, in urban or holiday regions, the number of patients who only once visit a pharmacy due to circumstances may be considerable. These would be falsely classified as nonadherent
14. “Actual patients” as exclusion criterion	Exclusion of non-actual patients	Inclusion of non-actual patients	Non-actual patients are subjects without a dispensing of any ATC drug class within the four last months at the end of the study period. As an example, patients who moved or died might falsely appear as nonadherent. These patients can be excluded as those without any dispensing in a pharmacy
15. Insulin users as exclusion criterion	Inclusion of subjects using insulin concomitantly to OHA	Exclusion of subjects using insulin concomitantly to OHA	T2DM users who cannot sufficiently be treated with three different OHA drug classes should receive insulin. They then may stop with all or some OHA drug classes. In this case, OHA discontinuation would be wrongly assumed as nonadherent. To avoid misclassification, insulin users could be excluded from nonadherence calculation. Use of insulin was defined by at least one dispensing of drug class A10A during the study period
16. Data source per pharmacy or pharmacy cluster	Individual community pharmacy	Clustered data from several community pharmacies	Valid assessment of patients’ adherence implies that all dispensings to a patient are taken into account. Using health-care claims databases, patient dispensings from several pharmacies are taken into account. However, when calculating patient’s adherence by data from one pharmacy only, dispensings from other pharmacies might be missed

^a^Methodological categories 1–12 were derived from Wilke et al. [7] and expanded by 4 categories relevant for OHA nonadherence measurement by pharmacy dispensing data

^b^Anatomic Therapeutic Chemical system of the World Health Organization

^c^Possible parameter values for variation not used here for the calculation of adherence measures are printed in italic

A basic scenario was formulated from all 16 categories. Each methodological category offered a choice of up to three parameter values. The choices in this scenario made were aimed to select patients nonadherent to OHA for additional pharmaceutical care and to prevent false-positive labeling for nonadherence (Table 1). The resulting basic case included all OHAs with at least one OHA dispensation during the study period (categories 1 and 3), as well as new users and those who stopped OHA use during the study period (categories 2 and 6), regardless of concomitant use of insulin (category 15) within the study year (category 4). For subjects who used several OHA classes concomitantly (category 10), PDC was calculated in three steps: (i) per patient as the medication possession rate for each OHA class, (ii) the arithmetic mean per patient of the different medication possession rates, and (iii) average of the arithmetic patient mean (category 7). Different OHA classes are used concomitantly in treatment of diabetes mellitus, so concomitant use of OHAs was considered in the adherence calculation instead of switching between drug classes (category 7). To prevent false-positive selection for nonadherence, only those patients that appeared to visit a particular pharmacy regularly (categories 11, 13, and 14) were included. Periods of drug use were calculated as PDC by dividing the number of dispensed OHA tablets by PDD (category 5). These periods were adjusted for early refills (category 8). To reflect nonadherence rather than overuse, adherence PDC scores were truncated at 100 % (category 9). For MRAP, a 80 % threshold was chosen, which corresponded to other OHA studies [25] (category 12). To assess the impact of these choices in the basic scenario on the three adherence measures, parameter values could be varied for nine methodological categories from our data (categories 2, 3, 7, 8, and 11–15).

### Analyses

A mean value was calculated for each adherence measure. The range of PDC scores for individual patients was shown by 5th and 95th percentiles. To examine further the validity of our assumptions, we undertook three subanalyses.

The first subanalysis determined if OHA cessation might have been due to insulin use (category 2b). For subjects with OHA use in the first 6 months of the study period but no OHA use in the final 6 months, it was investigated whether they used insulin after OHA cessation.

The second subanalysis explored whether users of one OHA class might have switched to another drug class (e.g., due to side effects) instead of using several OHA classes concomitantly (category 7). For OHA users in the first 6 months of the study period who started using another OHA class, whether they had ceased to use the earlier used OHA class was tested.

For the third subanalysis, the influence of clustered data that allowed follow-up of patients for their dispensations from different pharmacies (category 16) on adherence scores was assessed. This analysis could be done for pharmacies that had labeled themselves as pharmacy clusters in our database. Scores of adherence measures were compared with adherence scores calculated for individual pharmacies within these clusters.

## Results

For adherence assessments during the study period, data were available from 1737 of 1981 community pharmacies (88 %) in the Netherlands. For the basic scenario, 604,500 OHA users were included (Table 2). Mean PDC with OHA use was 88.3 % (5th percentile:95th percentile = 44.4 %:100 %) and on average 80.3 % of OHA users with a PDC ≥80 % (MRAP80). These data corresponded to a mean number of 69 nonadherent patients (MNNP80) per pharmacy. Variation of parameter values within the 16 methodological categories resulted in score changes for all three adherence measures (Table 2). Mean adherence scores ranged between 85.0 and 91.8 % for the PDC, between 75.3 and 86.1 % for MRAP80, and between 49 and 92 for the MNNP80 per pharmacy (Table 2). Adherence rates increased (with a corresponding decrease in the absolute number of nonadherent patients per pharmacy) for parameter choices that excluded new users (category 2a), excluded gaps for ceased use of OHA (category 2b), or excluded OHA users with less than two dispensations (category 3). Adherence scores also increased if gaps in the use of one OHA class were filled with periods of drug use from another drug class and, hence, switching between OHA classes was considered instead of concomitant use (category 7). Finally, adherence scores improved if users of insulin besides OHA use were excluded (category 15).

**Table 2:** Influence of variation in parameter values on adherence measures for oral hypoglycemic agents

Category^a^	Parameter choice	PDC		MRAP80		MNNP80 per pharmacy	Total number of included patients
		Mean PDC (5th; 95th percentile)	Difference to basic case	Mean MRAP	Difference to basic case		
	Basic case	88.3 (44.4; 100.0)	NA	80.3	NA	69	604,500
Effects of parameter values variation compared to the basic case choices, mutually exclusive							
2a	Only prevalent users, new users of oral hypoglycemic agents (OHA) are excluded	89.1 (50.9; 100.0)	+0.8	81.5	+1.2	55	513,290
2b	Users ceasing OHA use excluded	91.1 (58.6; 100.0)	+2.8	84.7	+4.4	50	567,704
3	Users with a minimum number of OHA dispensings <2 excluded	90.3 (55.9; 100.0)	+2.0	82.8	+2.5	57	576,293
7	Switching considered between OHA drug classes	91.8 (49.6; 100.0)	+3.5	86.1	+5.8	46	604,500
8	Stockpiling not considered	85.0 (41.9; 100.0)	−3.3	75.6	−4.7	85	604,438
11	Subjects with absence periods included	87.9 (42.7; 100.0)	−0.4	79.5	−0.8	72	611,702
13	Drop-in patients included	88.1 (43.0; 100.0)	−0.2	80.1	−0.2	70	610,444
14	Patients without actual drug use included	85.0 (28.5; 100.0)	−3.3	75.3	−5.0	92	649,004
15	Insulin users excluded	88.9 (47.4; 100.0)	+0.6	81.6	+1.3	49	465,454

Based on data from 1737 Dutch community pharmacies

^a^Numbering corresponding to the methodological categories introduced in Table 1

*PDC* percentage of days covered by medication, *MRAP80* mean rate of adherent patients with a PDC ≥80 %, *MNNP80 per pharmacy* mean number nonadherent patient at a PDC < 80 % per pharmacy, *NA* not applicable

Adherence rates decreased (with a corresponding increase in the absolute number of nonadherent patients per pharmacy) for choices of parameter values that did not consider stockpiling (category 4), included patients with periods of absence (category 5) or included drop-in patients (category 13) or included patients who did receive any drugs recently (category 14).

Variation of the MRAP80 threshold for a stricter boundary of a mean medication possession rate of ≥90 % (MRAP90) or a more liberal threshold of 60 % (MRAP60) to be regarded as adherent resulted in changes of MRAP scores to 69.2 % (MRAP90) and 90.8 % (MRAP60) compared with the basic scenario of 80.3 % (MRAP80) (category 12). This variation corresponded to a mean absolute number of 107 nonadherent patients (MNNP90) and 31 nonadherent patients (MNNP60) compared with 69 nonadherent patients per pharmacy (MNNP80).

The first subanalysis to ascertain if OHA cessation might have been due to insulin use (category 2b) involved 36,796 subjects with OHA use in the first 6 months of the study period and no use of OHAs in the final 6 months. From these 36,796 subjects, 3505 subjects (9.5 %) used insulin after OHA cessation. The other 33,291 subjects (90.5 %) did not receive insulin dispensations after OHA cessation. The second subanalysis (validity of the assumption for concomitant use of OHA classes instead of switching from one OHA class to another) involved 604,500 OHA users. From these 604,500 OHA users, 1932 subjects (0.3 %) were considered to be “switchers” due to starting a new OHA class after cessation of the previously used OHA class. For the third subanalysis (effect on adherence measures for following up patients for their dispensations across different pharmacies), 75 clusters were registered in the SFK database with 258 community pharmacies (13 % of all pharmacies) in the Netherlands, see supplementary information. On average, a cluster comprised 3.4 pharmacies. Following patients across different pharmacies increased all adherence scores for clustered data compared with data from individual pharmacies: for the basic scenario, PDC improved by 1.7 % for clustered data compared with dispensations within individual pharmacies, MRAP80 improved by 2.8 %, and the MNNP per pharmacy decreased by 19.

## Discussion

Overall adherence scores calculated by pharmacy dispensing data were relatively robust to variations in parameters within a methodological framework for the PDC and percentage of subjects with a PDC ≥80 % (MRAP80). Compared with a basic scenario defined by 16 methodological categories, different parameter choices within these categories changed the PDC by a maximum of 3.3 % and MRAP80 by a maximum of 5.8 %. These ranges were much lower than the nonadherence ranges of 16–97 % reported by Wilke et al. [7]. This variation was mainly due to whether or not information availability on the PDD. As the PDD was available in dispensing data, this reason for variation in adherence calculation was overcome by our data. However, numbers of OHA users per pharmacy were high, so these percentages corresponded with a substantial change (between 49 and 92) in the mean absolute numbers of nonadherent patients per pharmacy. The direction of changes in adherence scores from variations in parameter values were as expected from theoretical considerations. For instance, for MRAP, a variation in the threshold to label patients as “adherent” from at a PDC of 60, 80, or 90 % had a considerable influence on the scores of adherence measures. In the literature, a PDC of 80 % is commonly assumed to be sufficient to warrant the expected clinical outcomes. For OHA use, a PDC <80 % has been shown to be associated with an increased prevalence of hospitalization [26]. However, other cut-off points may be relevant depending on the specific drug treatment.

Another source of changes in adherence scores was the possibility of following patients according to their dispensations across different pharmacies by clustered data instead of using data per individual pharmacy. Use of dispensing data within individual pharmacies is a general limitation of pharmacy data. At present pooled data of different pharmacies can be used only in specific settings by pharmacies using the same anonymous codes for individual patients. If patients are likely to receive their medication from different pharmacies, the use of pooled data from pharmacy clusters can provide the individual pharmacists with more complete information on their patients’ drug use. As expected, use of clustered data improved the scores of adherence measures, though changes were moderate for the PDC and MRAP80, with improvement of ≈3 % for the basic scenario. However, for the basic scenario, the absolute numbers of patients identified for nonadherence decreased by on average 19 patients per pharmacy. This finding implies that, per pharmacy, 1 in 4 of the patients identified from data of individual pharmacies only were labeled falsely as being nonadherent due to dispensations from other pharmacies. It is probable that such pharmacies who registered as clusters shared their patient populations most with other community pharmacies and thus suffered most from incomplete information of their patients’ drug use. Hence, our findings from this subgroup might have overestimated the effects of clustered data for adherence calculation.

Another source of considerable variation shown by Wilke et al. addressed category 6 for calculations of drug use within dispensings versus calculations until the fixed end of an interval (e.g., 1 year) [7]. This criterion resulted in changes in adherence scores by more than 7 %. Adherence calculations between dispensations per definition exclude periods of drug discontinuation and so tend to overestimate adherence in clinical practice. As for pharmaceutical care patients who stop their treatment for a chronic disease also need to be identified, variation in a parameter value for adherence within dispensations was not considered as a relevant alternative in our analysis. Nevertheless, cessation of one or more OHA might be related to the start of insulin treatment, in which case the prescriber stopped OHA use and so an observed discontinuation might not reflect nonadherence. As a limitation of our study, we did not dispose of information on the reason for drug discontinuation. To assess the potential resulting bias, our subanalysis showed that only about 10 % of all subjects with OHA cessation used insulin subsequently. And even this limited percentage cannot be fully explained by prescriber-initiated OHA cessation as guidelines advise continuation of at least one OHA in combination with insulin. Our basic assumption to consider concomitant use rather than switching between different OHA classes was confirmed by a subanalysis which showed that only 0.3 % of OHA users in the first 6 months of the study period switched to another OHA class.

In conclusion, a framework with 16 methodological categories was useful to define OHA adherence based on pharmacy dispensing data. Adherence scores expressed as percentages were relatively robust to variations in parameter values but differed substantially in terms of the absolute numbers of nonadherent patients per pharmacy.

## Electronic supplementary material

ESM 1 (DOC 92 kb)
