# Supplementary material for: Use of pharmacy dispensing data to measure adherence and identify nonadherence with oral hypoglycemic agents
Source: Eur J Clin Pharmacol. 2016 Oct 28;73(2):205–13. doi: 10.1007/s00228-016-2149-3 (PMC5226973; doi:10.1007/s00228-016-2149-3)
Supplement: Supplementary file 1 — (DOC 92 kb) [file 228_2016_2149_MOESM1_ESM.doc]

# Supplementary information: Influence on adherence scores for following up dispensations across pharmacies compared with using dispensations within individual pharmacies onlya

|  | **Parameter choice** | **Patients’ dispensings followed across pharmacies** | | |  | **Patients’ dispensings within individual pharmacies only** | | | |
| --- | --- | --- | --- | --- | --- | --- | --- | --- | --- |
| **Category^b^** |  | **PDC^c^ (5^th^; 95^th^ percentile)** | **MRAP80^d^** | **MMNP80^e^** | **Total number included patients** | **PDC^c^ (5^th^ ; 95^th^ percentile)** | **MRAP80^d^** | **MNNP80^e^** | **Total number included patients** |
|  | **Basic case** | **89.3 (49.6; 100.0)** | **82.0** | **59** | **84,219** | **87.6 (40.2;100.0)** | **79.2** | **78** | **96,718** |
| **Effects of parameter values variation compared to the basic case choices, mutually exclusive** | | | | | | | | | |
| 2a | Patient type: starters excluded | 89.8 (54.5; 100.0) | 82.7 | 50 | 74,059 | 88.6 (48.5;100.0) | 80.6 | 61 | 81,382 |
| 2b | Patient type: stoppers excluded | 91.7 (60.4; 100.0) | 85.8 | 44 | 79,744 | 90.8 (56.8;100.0) | 84.0 | 56 | 90,238 |
| 3 | Minimum number of prescriptions: 2 | 90.8 (57.0; 100.0) | 84.0 | 51 | 81,410 | 89.9 (54.3;100.0) | 82.1 | 64 | 91,633 |
| 7 | Switching considered between OHA^f^ drug classes | 92.7 (55.1; 100.0) | 88.3 | 38 | 84,219 | 91.1 (44.2;100.0) | 85.7 | 54 | 96,718 |
| 8 | Stockpiling: not considered | 86.0 (46.9; 100.0) | 77.5 | 73 | 84,210 | 84.5 (38.4;100.0) | 74.7 | 95 | 96,706 |
| 11 | Subjects with absence periods included | 89.0 (48.6; 100.0) | 81.4 | 61 | 84,844 | 87.1 (38.5;100.0) | 78.2 | 83 | 98,090 |
| 13 | Drop in patients included | 89.2 (49.3; 100.0) | 81.9 | 59 | 84,510 | 87.5 (39.3;100.0) | 79.1 | 79 | 97,568 |
| 14 | Patients without actual drug use included | 87.3 (37.2; 100.0) | 78.8 | 72 | 87,957 | 84.3 (25.6;100.0) | 74.2 | 104 | 103,948 |
| 15 | Insulin users excluded | 90.1 (52.6; 100.0) | 83.6 | 41 | 64,140 | 88.3 (43.2;100.0) | 80.6 | 56 | 74,489 |

^a^ Based on numbers of 258 Dutch community pharmacies within 75 clusters / ^b^ Numbering corresponding to the methodological categories introduced in Table 1.

^c^ Percentage of days covered by medication / ^d^ MRAP80 mean rate of adherent patients with a PDC ≥80% / ^e^ Mean number non adherent patient at a PDC<80% per pharmacy/ ^f^ oral hypoglycaemic agents
